# Long noncoding RNA MSC‐AS1 promotes hepatocellular carcinoma oncogenesis via inducing the expression of phosphoglycerate kinase 1

**DOI:** 10.1002/cam4.3080

**Published:** 2020-06-02

**Authors:** Cong Cao, Qiuhong Zhong, Liuxue Lu, Bin Huang, Jun Li, Lianxin Meng, Hua Wei

**Affiliations:** ^1^ Department of General Practice The Affiliated Hospital of Youjiang Medical University for Nationalities Baise Guangxi Province China; ^2^ Nursing Department The Affiliated Hospital of Youjiang Medical University for Nationalities Baise Guangxi Province China; ^3^ Department of Ultrasonics The Affiliated Hospital of Youjiang Medical University for Nationalities Baise Guangxi Province China

**Keywords:** cell viability, hepatocellular carcinoma, invasion, MSC‐AS1, PGK1

## Abstract

**Background and Objectives:**

Increasing studies report that lncRNAs are dysregulated in hepatocellular carcinoma (HCC), which might function as significant diagnostic biomarkers of HCC. LncRNA MSC‐AS1 has been newly discovered in several cancers. However, its biological effect in HCC remains to be clearly elucidated. The aim of our work was to test MSC‐AS1 expression level and assess its function in HCC progression.

**Methods:**

Expression levels of MSC‐AS1 and PGK1 in HCC were tested by qRT‐PCR in HCC cells including HUH‐7, BEL‐7404, SNU449, HepG2, QGY‐7701, and human normal liver cells (HL‐7702 cells). Association of MSC‐AS1 expression with various clinicopathological features and patients’ survival were analyzed by chi‐squared test and Kaplan‐Meier, respectively. The functions of MSC‐AS1 in HCC cells were investigated using EdU assay, colony formation, flow cytometry, would healing assay, and Transwell matrigel invasion assays. The correlation between MSC‐AS1 and PGK1 was confirmed using a RIP assay. Protein expression of PGK1 was evaluated using a western blot assay.

**Results:**

MSC‐AS1 was obviously upregulated in HCC tissues and HCC cells. Knockdown of MSC‐AS1 repressed HepG2 and BEL‐7404 cell proliferation, colony formation capacity, and triggered cell apoptosis. HepG2 and BEL‐7404 cell cycle was blocked in G1 phase and cell migration/invasion was remarkably depressed. Downregulation of MSC‐AS1 in HCC cells reduced PGK1 expression. In vivo data demonstrated that silence of MSC‐AS1 suppressed HCC development via activating PGK1.

**Conclusions:**

Taken these together, we indicated that MSC‐AS1 promoted HCC oncogenesis via inducing the expression of PGK1.

## Graphical abstract

MSC‐AS1 manifested an oncogenic role in HCC. MSC‐AS1 could contribute to HCC progression through activating PGK1 in HCC.

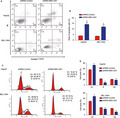

## 1 INTRODUCTION

Hepatocellular carcinoma (HCC) is a great cause of cancer‐related mortality worldwide.^1^, ^2^ Every year, more than 500,000 new cases are reported.^3^ In spite of the advances made in the therapy of HCC, it can still cause half a million deaths each year.^4^, ^5^ Hence, a detailed understanding of the mechanisms in HCC is needed to develop its effective therapies.

Increasing estimations have suggested that almost 75% human genome is transcribed, which can generate various noncoding RNAs (ncRNAs).^6^ The majority of these ncRNAs have the length over 200 nts and they are defined as lncRNAs.^7^ It has been shown that lncRNAs can participate in regulating gene expression, chromatin remodeling, controlling transcription, regulating splicing, stabilizing mRNA, microRNA processing, and maintaining protein stability.^8^ In addition, lncRNAs appear to exhibit major roles in various diseases.^9^, ^10^, ^11^

Accumulating lncRNAs are reported to be involved in HCC. For example, lncRNA MALAT1 can promote HCC progression through upregulating SRSF1 and the activation of mTOR.^12^ Lnc‐EGFR can stimulate differentiation of T‐regulatory cells, which can promote HCC immune evasion.^13^ HOXA11‐AS induces HCC cell proliferation via modulating LATS1.^14^ In addition, lncRNA CASC2 represses HCC cell epithelial‐mesenchymal transition (EMT) via regulating CASC2/miR‐367/FBXW7.^15^ It has been indicated that MSC‐AS1 can modulate pancreatic ductal adenocarcinoma cell proliferation and apoptosis through sponging miR‐29b‐3p.^16^ Nevertheless, the specific effect of MSC‐AS1 in HCC are not fully explained.

Phosphoglycerate kinase 1 (PGK1) is an important essential enzyme in aerobic glycolysis. PGK1 is closely involved in various biological activities, such as angiogenesis, autophagy, and DNA repair.^17^ In addition, the function of PGK1 in cancer progression is complicated and has been reported in recent years.^18^ However, the detailed roles of PGK1 in HCC remain poorly known.

Currently, we investigated the biological roles of MSC‐AS1 in HCC. MSC‐AS1 and PGK1 were greatly increased in HCC. Additionally, loss of MSC‐AS1 inhibited HCC via inducing PGK1 expression. Hence, we hypothesized that MSC‐AS1 could repress HCC development through regulating PGK1.

## 2 MATERIALS AND METHODS

### 2.1 Clinical samples

Sixty HCC tissue and paired adjacent noncancerous tissue samples were obtained from our hospital. Our study selected 60 cases (gender: 44 males and 16 females; age: 40‐70 years; mean: 49.8 ± 4.1 years). No therapies for any clinical disorders were carried out within 100 days and there were no other clinical disorders or recurrent HCC. The samples were maintained in liquid nitrogen after surgery. Diagnosis of HCC was verified pathologically. The clinicopathological characteristics of HCC patients were exhibited in Table 1. No patients were given radiotherapy or chemotherapy before the surgery. Before we collected the samples, all the patients provided the written informed consent. The study was approved by the local research Ethics Committee.

**Table 1:** Clinicopathological relevance analysis of MSC‐AS1 from HCC tissue samples

Feather	Patients	MSC‐AS1		*P* value
		Low expression (<median)	High expression (≥median)	
All cases	60	30	30	
Age, y				.419
<60	39	17	22	
≥60	21	13	8	
Gender				.661
Male	44	23	21	
Female	16	7	9	
Tumor size (cm)				**.003**
≤5	36	24	12	
>5	24	6	18	
Tumor number				.432
Solitary	40	25	15	
Multiple	20	5	15	
TNM stage (I: II: III)				**.003**
I	22	18	4	
II	16	5	11	
III	22	7	15	
Metastasis				**.001**
Yes	21	9	12	
No	39	21	18	

Note

We analyzed the data from 60 HCC patients. For MSC‐AS1 level, the median expression served as the cutoff. Data were analyzed using chi‐squared test. *P*‐value in bold suggested the statistically significance.

### 2.2 Cell culture

HCC cells (HUH‐7, BEL‐7404, SNU449, HepG2, and QGY‐7701 cells) and human normal liver cells (HL‐7702 cells) were obtained from the Cell Bank of the Chinese Academy of Sciences. Cells were cultured in RPMI1640 medium with 10% FBS (GIBCO), 100 U/ml penicillin, and 100 mg/mL streptomycin (Invitrogen) at 37°C with 5% CO_2_.

### 2.3 Lentivirus infection

shRNA was designed to target MSC‐AS1 in HCC cells. shRNA‐MSC‐AS1 control shRNA lentivirus particles were obtained from GenePharma. 293T cells were used for retroviral packaging. HepG2 and BEL‐7404 cells were treated with virus‐containing supernatant with 8 mg/mL polybrene. After 48 hours, puromycin was used to select the infected cells. Cells were transduced with the lentiviruses at multiplicity of infection (MOI) of 2 for 72 hours. The expression of MSC‐AS1 in the infected cells was tested using qRT‐PCR.

### 2.4 EdU proliferation

Cells were seeded into 96‐well plates. After incubation, each well was added with 50 μmol/L EdU solution. Apollo staining and DAPI staining were employed to observe EdU‐positive cells under a fluorescence microscope.

### 2.5 Colony formation analysis

Cells were grown into 10 cm plates and then, incubated for 14 days. Cells were fixed using methanol, stained by Crystal violet solution (Sigma). Colony numbers were counted using Photoshop software.

### 2.6 Wound healing assay

Cells were seeded into the six‐well plates for a whole night. To create a cell‐free clear zone, we scratched the confluent monolayer using a plastic apparatus. Then, the cells were incubated in the medium without FBS. The wound distance was tested regularly.

### 2.7 Matrigel invasion assay

Matrigel invasion chamber was employed to examine cell invasion ability. Briefly, cells were grown in the upper chamber with the media with 0.1% BSA. The media with 30% FBS was added in the lower well. Then, we removed the noninvading cells using cotton swabs. Afterward, invasive cells remaining at bottom were fixed by 1% formalin and stained by 0.1% crystal violet. We counted the cells using microscopic observation.

### 2.8 Analysis of cell apoptosis

The transfected cells were harvested and washed using PBS. The double staining with 0.5 mg/mL FITC‐Annexin V and 5 mg/mL PI was carried out for half an hour at room temperature using the Annexin V/PI Detection Kit (BD Biosciences). We counted and compared the percentage of the cells in different phases. We analyzed the cells using a flow cytometry and a Cell Quest 3.0 software.

### 2.9 Flow cytometric analysis of cell cycle

Cell cycle was assessed using flow cytometric analysis. After treatment, cells were collected by trypsinization and then fixed in 70% ethanol for a whole night. Next, the fixed cells were rehydrated in PBS and digested using RNase A and labeled with PI. Data were analyzed by Flow Jo analysis software.

### 2.10 Western blotting

To carry out western blotting analysis, 40 g protein of each sample was separated on 10% SDS‐PAGE. Then, the protein was transferred to PVDF membranes (Millipore, Billerica). After blocking using 5% milk in PBS‐0.05% Tween, they were incubated with the primary antibody at 4°C. Subsequently, the membranes were incubated with secondary antibodies and finally detected using ECL substrate kit (Tanon). Primary antibody against GAPDH and PGK1 and HRP‐conjugated secondary antibodies were purchased from Cell Signaling Technology.

### 2.11 qRT‐PCR

Total RNA was extracted by TRIzol reagent (Invitrogen). Isolated RNA was tested by OD260/280 using Nanodrop (Thermo Scientific). RNA was reverse‐transcribed into cDNA by a PrimeScript RT reagent Kit (TaKaRa Bio Technology). To quantify the expression levels of MSC‐AS1 and PGK1, quantitative PCR was conducted using the SYBR kit (BioRad) on a 7900 Fast Real‐Time PCR system (Applied Biosystems). Primers were manifested as follows:

GAPDH forward 5′‐ CTCAGACACCATGGGGAAGGTGA −3′.

and reverse 5′‐ATGATCTTGAGGCTGTTGTCATA‐3′;

MSC‐AS1 forward 5′‐GCCAGTCAGAAAATGAGGAAC‐3′.

and reverse 5′‐ CCAGTTGGGTGAACAGGAC −3′;

PGK1 forward 5′‐ ACTCGGGCTAAGCAGATTGT −3′.

and reverse 5′‐ GTGCTCACATGGCTGACTTT −3′.

The relative quantitative data were analyzed by the 2^−ΔΔCt^.

### 2.12 RIP assay

To test whether MSC‐AS1 was associated with PGK1, RIP assays were conducted using an EZMagna RIP Kit (Millipore, Billerica) and a PGK1 antibody (Santa Cruz Biotechnology). IgG (Millipore, Billerica) served as a negative control. Subsequently, qRT‐PCR analysis was carried out to measure the expression levels of MSC‐AS1 and PGK1.

### 2.13 Animal studies

Twelve female BALB/c mice aged 5‐6 weeks and weighed 16‐18 g (Shanghai Animal Laboratory Center) were maintained in the Experimental Animal Center. HepG2 cells infected with shRNA‐control or MSC‐AS1 shRNA were injected into the mice in the right flanks of nude mice. Every three days, tumor sizes were recorded. After 6 weeks postinjection, animals were sacrificed by cervical dislocation. Then, the tumors were exercised and weighted. Our study was performed under the Guide for the Care and Use of Laboratory Animals of the National Institutes of Health.

### 2.14 Statistical analysis

Student's *t* tests and one‐way analysis of variances with Tukey's multiple comparisons tests were used to analyze the significance of differences between groups. Correlations between MSC‐AS1 and PGK1 were analyzed using linear regression. We established survival curves by the Kaplan‐Meier method. A value of *P* < .05 was considered to be as statistically significant. GraphPad Prism 6 statistical package (GraphPad Inc) and SPSS 18.0 software package (SPSS Inc) were used to do statistical analysis.

## 3 RESULTS

### 3.1 Expression of MSC‐AS1 and PGK1 was increased in HCC

**Figure 1:**
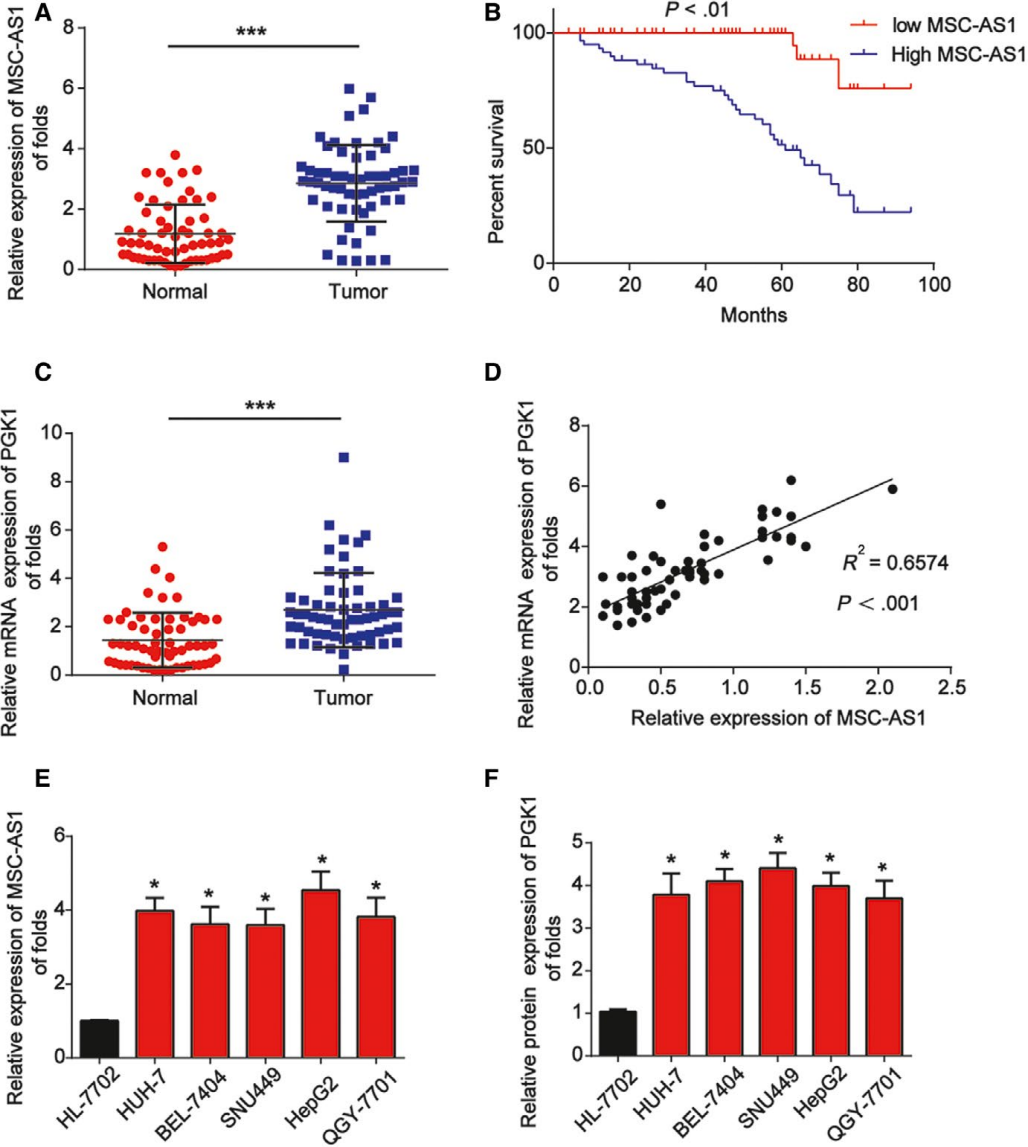
Expression of MSC‐AS1 and PGK1 in HCC specimens. A, MSC‐AS1 expression in HCC tissues compared with paired noncancerous tissues (n = 60). B, Kaplan‐Meier analysis of progression‐free survival based on MSC‐AS1 levels in 60 HCC patients. C, PGK1 mRNA expression in HCC tissues compared with paired noncancerous tissues (n = 60). D, Correlation between expression of MSC‐AS1 and PGK1 in tissue specimens. E, MSC‐AS1 expression in HUH‐7, BEL‐7404, SNU449, HepG2, QGY‐7701, and HL‐7702 cells. F, PGK1 mRNA in HUH‐7, BEL‐7404, SNU449, HepG2, QGY‐7701, and HL‐7702 cells. Three independent experiments were carried out. Error bars stand for the mean ± SD of at least triplicate experiments. ***P < .001

First, to test MSC‐AS1 expression in HCC, 60 pairs of HCC tumors and the corresponding adjacent tissues were collected. As exhibited in Figure 1A, we observed that MSC‐AS1 was obviously elevated in HCC tissues. Kaplan‐Meier survival curves indicated that HCC patients with high MSC‐AS1 levels displayed a worse progression‐free survival (Figure 1B). Then, PGK1 expression was determined and we found it was also greatly upregulated in HCC tissue samples (Figure 1C). In HCC tissues, a positive correlation between MSC‐AS1 and PGK1, which was analyzed using Spearmen's correlation, was observed, supporting the role of MSC‐AS1 in the expression of PGK1 in Figure 1D. The expression of MSC‐AS1 and PGK1 mRNA expression were tested in HCC cells (HUH‐7, BEL‐7404, SNU449, HepG2, and QGY‐7701 cells) and human normal liver cells (HL‐7702 cells). We observed that MSC‐AS1 and PGK1 expression was greatly increased in HCC cells compared to HL‐7702 cells as displayed in Figure 1E,F.

### 3.2 Loss of MSC‐AS1 repressed HCC cell proliferation

**Figure 2:**
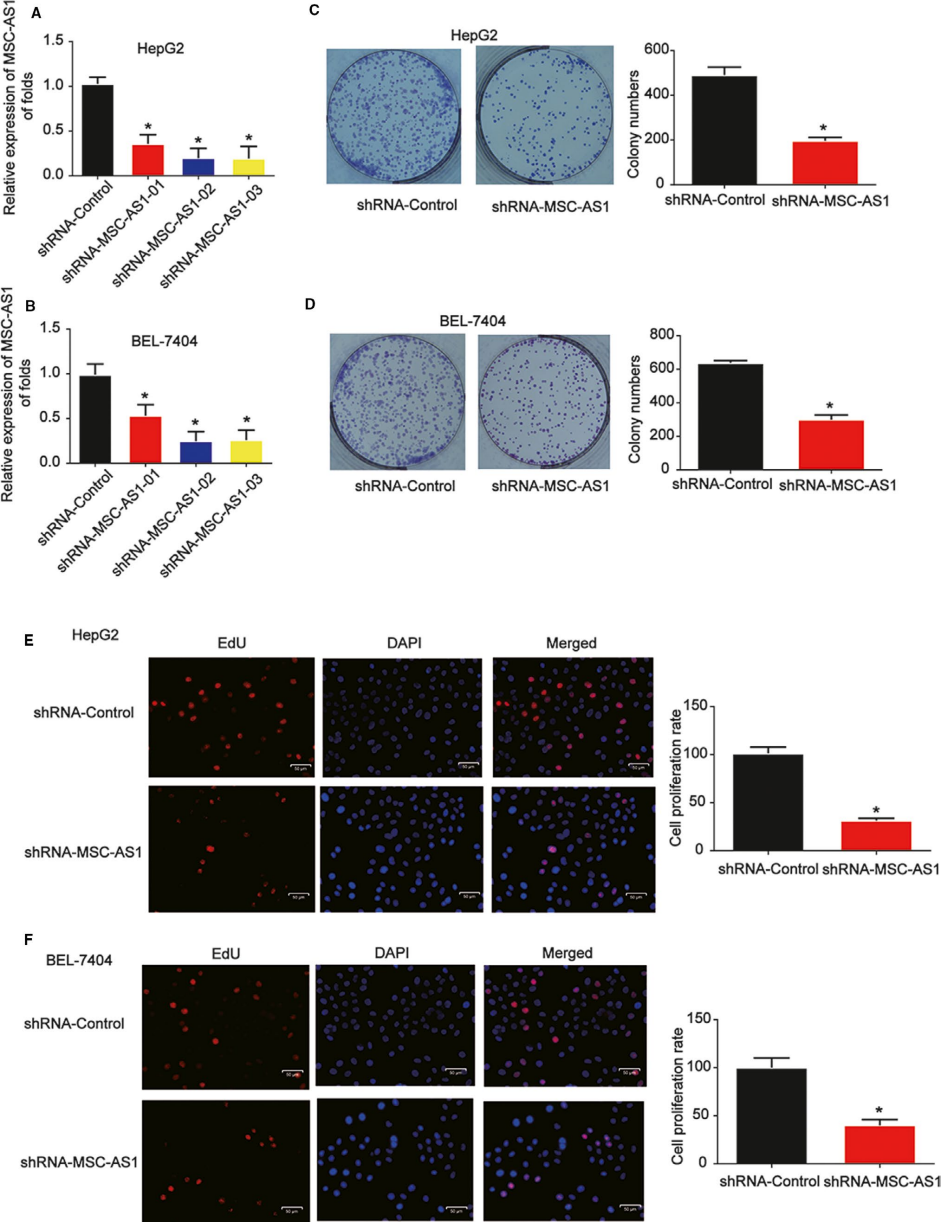
Effects of MSC‐AS1 on HCC cell proliferation. A and B, MSC‐AS1 expression in HepG2 and BEL‐7404 cells. Cells were infected with shRNA of MSC‐AS1 for 48 h. C and D, Effects of MSC‐AS1 shRNA on cell colony formation. E and F, Effects of MSC‐AS1 shRNA on cell proliferation. Three independent experiments were carried out. Error bars stand for the mean ± *SD* of at least triplicate experiments. *P < .05

Furthermore, we established MSC‐AS1 knockdown HCC cell lines using HepG2 and BEL‐7704 cells. Decreased expression of MSC‐AS1 in cells infected with MSC‐AS1 shRNA was confirmed using qRT‐PCR. In Figure 2A,B MSC‐AS1 shRNA 02 exhibited a best knockdown effect in HCC cells. To carry out the subsequent experiments, MSC‐AS1 shRNA 02 was utilized to inhibit MSC‐AS1 expression. In order to find out whether MSC‐AS1 can influence HCC cell proliferation, colony formation, and EdU assay were carried out. HepG2 and BEL‐7704 cell colony formation was repressed by MSC‐AS1 shRNA (Figure 2C,D). Meanwhile, EdU assay indicated that HepG2 and BEL‐7704 cell proliferation was obviously inhibited by decrease of MSC‐AS1 (Figure 2E,F).

### 3.3 Downregulated MSC‐AS1 enhanced HCC cell apoptosis and blocked cell cycle progression

**Figure 3:**
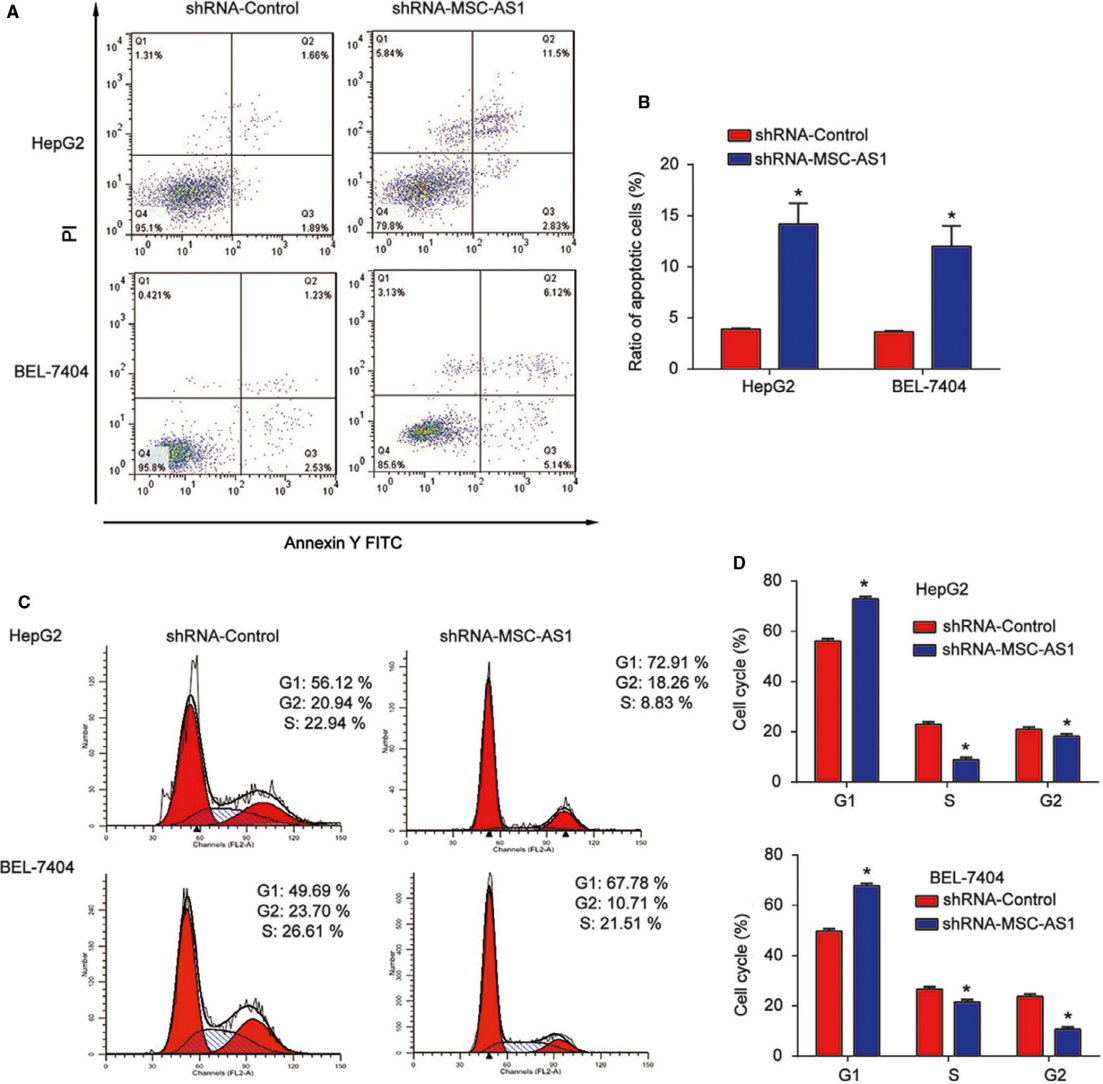
Effects of MSC‐AS1 on HCC cell apoptosis and cell cycle progression. A and B, Effects of MSC‐AS1 shRNA on HepG2 and BEL‐7704 cell apoptosis. Flow cytometry assay was employed to analyze cell apoptosis. C and D, Effects of MSC‐AS1 shRNA on HepG2 and BEL‐7704 cell cycle. Three independent experiments were carried out. Error bars stand for the mean ± *SD* of at least triplicate experiments. *P < .05

Next, flow cytometry was conducted to analyze the effect of MSC‐AS1 on HCC cell apoptosis and cell cycle. We proved apoptosis of HCC cells was greatly induced by MSC‐AS1 shRNA as shown in Figure 3A,B. In addition, HCC cell cycle was significantly arrested in G1 phase as manifested in Figure 3C,D.

### 3.4 Knockdown of MSC‐AS1 depressed HCC cell migration and invasion

**Figure 4:**
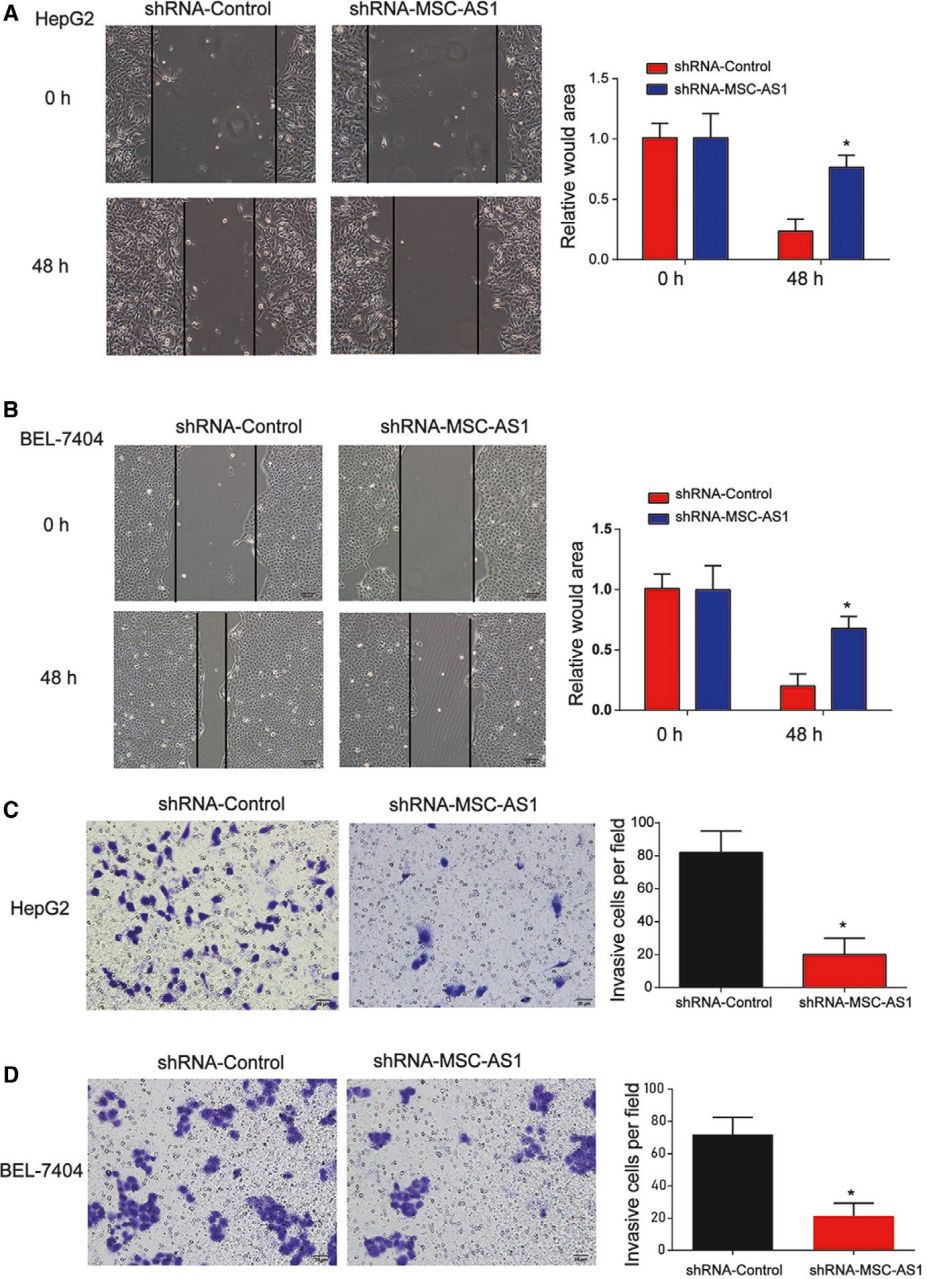
Effects of MSC‐AS1 on HCC cell migration and invasion. A and B, Effects of MSC‐AS1 shRNA on HepG2 and BEL‐7704 cell migration. Would healing assay was carried out to test cell migration ability. C and D, Effects of MSC‐AS1 shRNA on HepG2 and BEL‐7704 cell invasion. Transwell invasion was performed. Three independent experiments were carried out. Error bars stand for the mean ± SD of at least triplicate experiments. *P < .05

Furthermore, would healing assay was carried out in Figure 4A,B and we found HepG2 and BEL‐7704 cell migration was greatly restrained by loss of MSC‐AS1. In addition, Transwell invasion was carried out and HCC invasion capacity was repressed by the downregulation of MSC‐AS1 as shown in Figure 4C,D.

### 3.5 MSC‐AS1 interacted with PGK1

**Figure 5:**
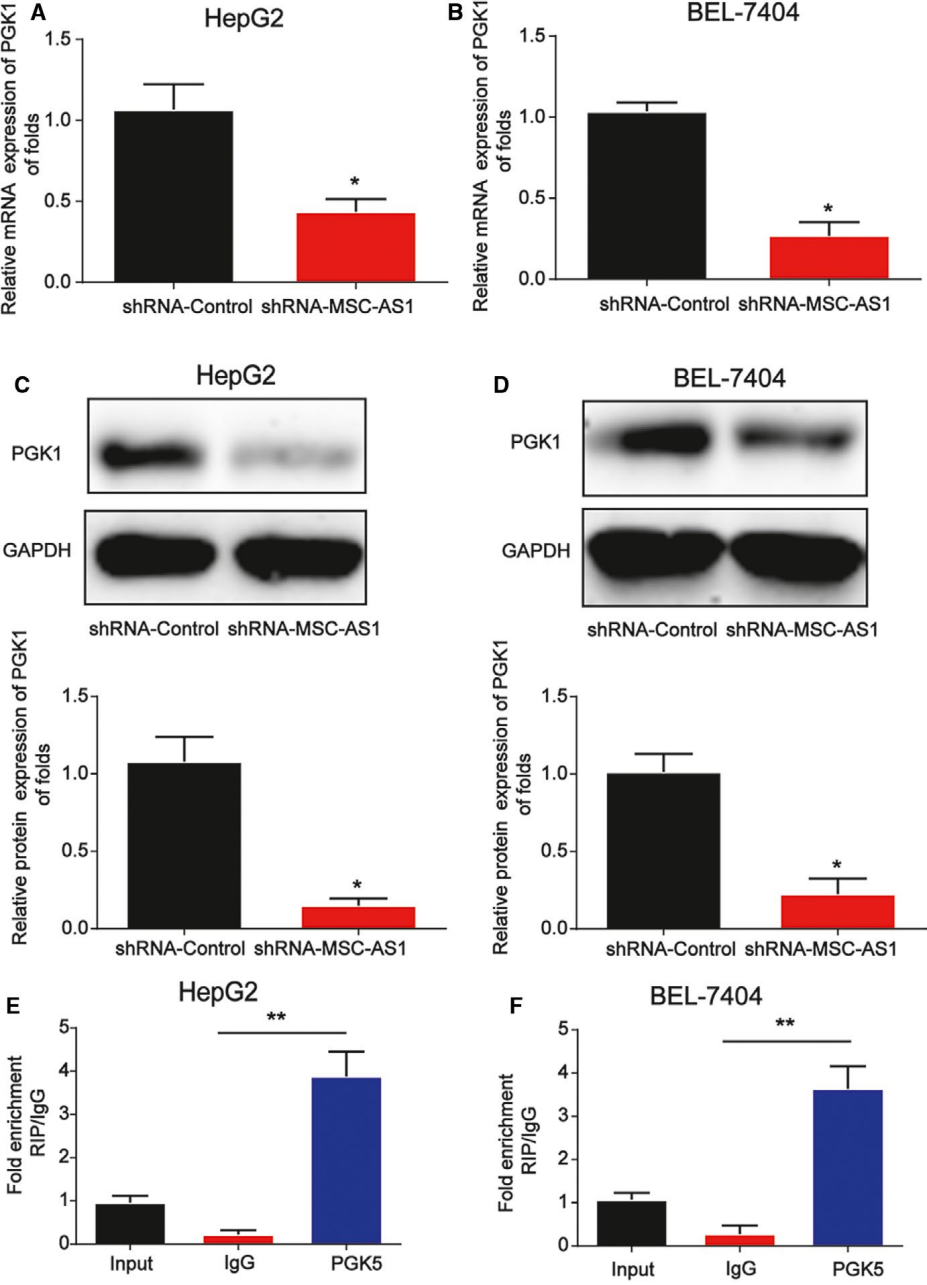
PGK1 interacted with MSC‐AS1. A and B, PGK1 mRNA expression in HepG2 and BEL‐7404 cells. Cells were infected with shRNA of MSC‐AS1 for 48 h. C and D, PGK1 protein expression in HepG2 and BEL‐7404 cells. E and F, The enrichment ratio of MSC‐AS1 with PGK1 antibody compared with IgG, as determined by a RIP assay in HepG2 and BEL‐7404 cells. Three independent experiments were carried out. Error bars stand for the mean ± *SD* of at least triplicate experiments. *P < .05

In Figure 5A,B, PGK1 mRNA level was repressed by loss of MSC‐AS1. Consistently, PGK1 protein level exhibited a similar tendency as shown in Figure 5C,D. Then, the RIP assay was performed using anti‐PGK1 in HCC cells to confirm the relationship between PGK1 and MSC‐AS1. These revealed that MSC‐AS1 immunoprecipitated with PGK1 antibody was increased relative to IgG control (Figure 5E,F).

### 3.6 **Downregulation of MSC‐AS1 inhibited HCC progression in vivo via interacting with PGK**1

**Figure 6:**
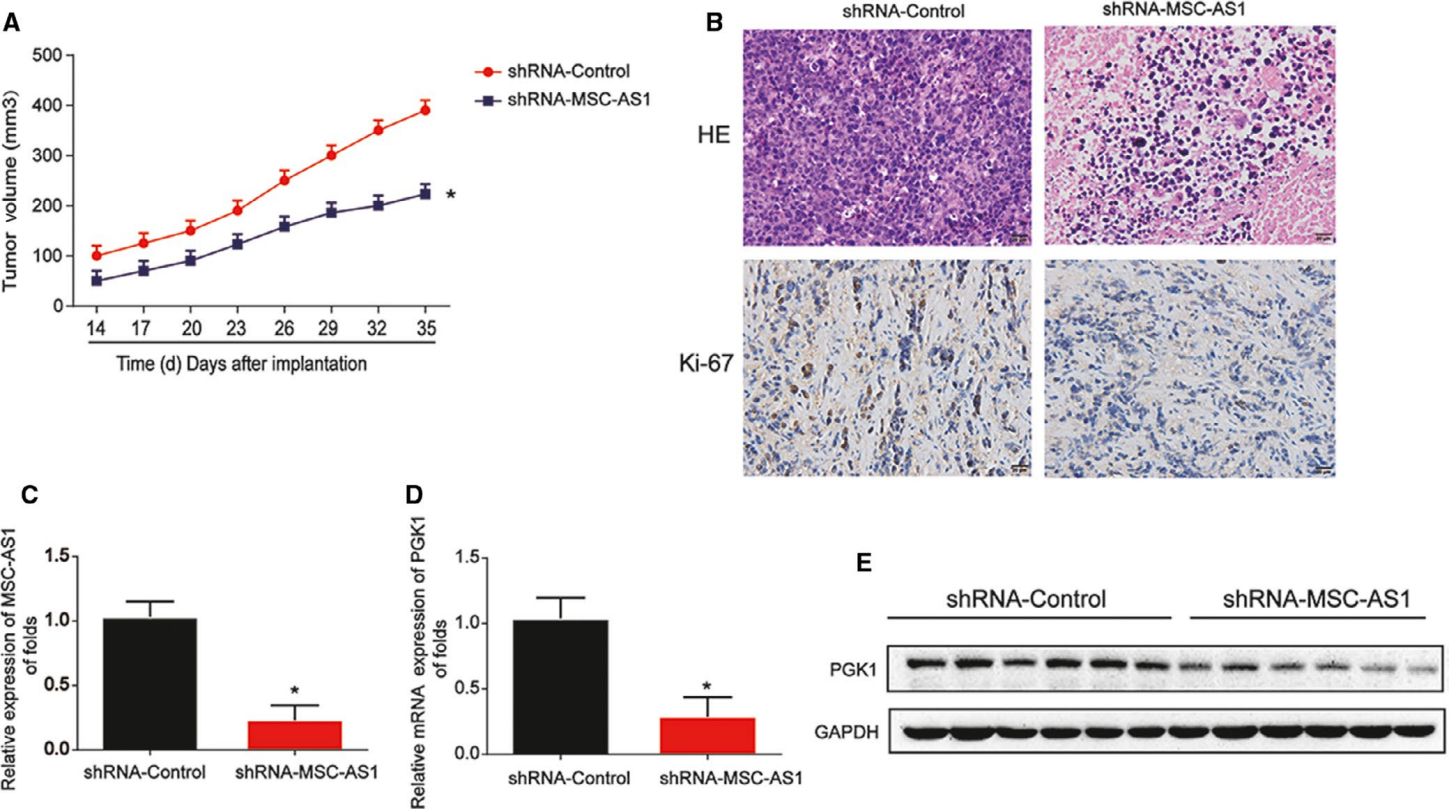
Loss of MSC‐AS1 depressed HCC progression via inactivating PGK1 in vivo. Twelve 8‐week‐old female BALB/c nude mice were injected with HepG2 cells infected with shRNA‐control (six mice) or shRNA‐MSC‐AS1 (six mice). A, Tumor volume in a time‐dependent manner. B, H＆E staining and IHC staining of Ki‐67 in tumor tissues. C, Expression of MSC‐AS1 in the tumor tissues. D and E, Expression of PGK1. Three independent experiments were carried out. Error bars stand for the mean ± *SD* of at least triplicate experiments. *P < .05

HepG2 cell nude mouse xenograft model was set to study whether loss of MSC‐AS1 can repress HCC progression. Mice were injected with HepG2 cells infected with shRNA‐control or MSC‐AS1. As demonstrated in Figure 6A, tumor volume was repressed in a time‐dependent course. In addition, HE and IHC data was implied in Figure 6B. Meanwhile, we proved that knockdown of MSC‐AS1 greatly depressed PGK1 expression in vivo (Figure 6C‐E).

## 4 DISCUSSION

Recently, the recognition of lncRNAs has indicated a new understanding of disease pathogenesis. LncRNAs are RNA genes and they can regulate gene expression through various mechanisms such as interacting with RNAs or protein molecules. Up to now, many lncRNAs have been identified as crucial biomarkers in HCC.^19^, ^20^, ^21^ For instance, lncRNA HULC can induce autophagy through stabilizing Sirt1, which reduce the chemosensitivity of HCC cells.^22^ Loss of lncRNA ANRIL depresses HCC progression through regulating miR‐122‐5p.^23^ In addition, lncRNA TP73‐AS1 regulates HCC cell proliferation through the modulation of miR‐200a and HMGB1/RAGE.^24^

Here, we reported MSC‐AS1 was significantly increased in HCC. Inhibition of MSC‐AS1 greatly restrained HCC progression in vitro. Additionally, we found PGK1 was also remarkably upregulated in HCC, which was positively correlated with MSC‐AS1. Finally, the in vivo experiments were conducted and the data exhibited loss of MSC‐AS1 depressed HCC development through modulating PGK1.

LncRNA MSC‐AS1 has been studied in recent years. For example, MSC‐AS1 can promote the BMSCs osteogenic differentiation via repressing miR‐140‐5p to induce BMP2, which can alleviate osteoporosis progression.^25^ A previous study has reported MSC‐AS1 predicts the recurrence‐free survival of HCC.^26^ Currently, we found MSC‐AS1 was upregulated in HCC and its high expression indicated a bad progression‐free survival. Then, we observed that loss of MSC‐AS1 greatly inhibited HCC progression.

PGK1 is a significant oncogene in various cancers.^27^, ^28^, ^29^ For example,miR‐548c‐5p represses colorectal cancer cell proliferation through targeting PGK1.^30^ PGK1 can act as a protein kinase to regulate glycolysis and TCA Cycle in carcinogenesis.^31^ PGK1 is a survival biomarker for breast cancer and it can promote invasion via modulating the EMT process.^32^ Acetylation of PGK1 can induce liver cancer tumorigenesis.^18^ In addition, miR‐450b‐3p represses HCC cell growth through inhibiting PGK1.^33^ Consistently, we proved that PGK1 was increased in HCC specimens. For another, we found a positive correlation between PGK1 and MSC‐AS1. Further assays revealed that MSC‐AS1 repressed HCC progression via interacting with PGK1. Whether PGK1 could act as a direct or indirect target of MSC‐AS1 will be investigated in our future study.

In conclusion, we reported in our work that MSC‐AS1 manifested an oncogenic role in HCC. A potential interaction between MSC‐AS1 and PGK1 was implied in HCC progression. Our data suggested that MSC‐AS1 could contribute to HCC progression through activating PGK1 in HCC.

## CONFLICT OF INTEREST

The authors declare that they have no conflict of interest.

## Data Availability

The datasets used during the current study are available from the corresponding author on reasonable request.
